# Evaluation of the Usefulness of Topological Indices for Predicting Selected Physicochemical Properties of Bioactive Substances with Anti-Androgenic and Hypouricemic Activity

**DOI:** 10.3390/molecules28155822

**Published:** 2023-08-02

**Authors:** Dawid Wardecki, Małgorzata Dołowy, Katarzyna Bober-Majnusz

**Affiliations:** 1 Faculty of Pharmaceutical Sciences in Sosnowiec, Doctoral School, Medical University of Silesia in Katowice, 41-200 Sosnowiec, Poland; 2 Department of Analytical Chemistry, Faculty of Pharmaceutical Sciences in Sosnowiec, Medical University of Silesia in Katowice, 41-200 Sosnowiec, Poland; bober@sum.edu.pl

**Keywords:** anti-androgens, cluster analysis, hypouricemic agents, quantitative structure properties relationships, logP, lipophilicity, principal component analysis, R_MW_, topological indices

## Abstract

Due to the observed increase in the importance of computational methods in determining selected physicochemical parameters of biologically active compounds that are key to understanding their ADME/T profile, such as lipophilicity, there is a great need to work on accurate and precise in silico models based on some structural descriptors, such as topological indices for predicting lipophilicity of certain anti-androgenic and hypouricemic agents and their derivatives, for which the experimental lipophilicity parameter is not accurately described in the available literature, e.g., febuxostat, oxypurinol, ailanthone, abiraterone and teriflunomide. Therefore, the following topological indices were accurately calculated in this paper: Gutman (M, M^ν^), Randić (^0^χ, ^1^χ, ^0^χ^ν^, ^1^χ^ν^), Wiener (W), Rouvray–Crafford (R) and Pyka (A, ^0^B, ^1^B) for the selected anti-androgenic drugs (abiraterone, bicalutamide, flutamide, nilutamide, leflunomide, teriflunomide, ailanthone) and some hypouricemic compounds (allopurinol, oxypurinol, febuxostat). Linear regression analysis was used to create simple linear correlations between the newly calculated topological indices and some physicochemical parameters, including lipophilicity descriptors of the tested compounds (previously obtained by TLC and theoretical methods). Our studies confirmed the usefulness of the obtained linear regression equations based on topological indices to predict ADME/T important parameters, such as lipophilicity descriptors of tested compounds with anti-androgenic and hypouricemic effects. The proposed calculation method based on topological indices is fast, easy to use and avoids valuable and lengthy laboratory experiments required in the case of experimental ADME/T studies.

## 1. Introduction

Until now, the discovery and development of new, effective and safe drugs and formulations has been a very lengthy and costly process. Among different stages, the most important part of the whole drug discovery procedure is the optimization of ADME/T properties of a drug [1,2]. ADME/T studies have become increasingly important to help scientists identify which drug candidates are the most promising and can be prepared for clinical trials [2]. Despite the discovery of millions of new molecules in recent years, the number of new drugs has not increased significantly. The reason is the inadequate ADME/T profile of newly developed molecules. The processes in question are absorption, distribution, metabolism, elimination and toxicity of the drug. The right parameters determine the effectiveness of the drug, which can be explained by the appropriate level of binding to proteins or the length of time it remains in the patient’s bloodstream [1,2,3]. For this reason, ADME/T studies are now routinely performed at the early stage of a molecule’s discovery, as the results determine the further fate of the drug candidate and its commercial product next. The ADME/T tests allow for the examination of many factors affecting the action and effectiveness of the drug. Due to the growing importance of these studies, different methods for assessing ADME/T properties have been developed including modern methods supported by different analytical methods, such as chromatography in combination with mass spectrometry or NMR spectroscopy [3]. Usually, such studies are expensive, time consuming and require animal trials to conduct experimentally. Thanks to the intensive development of chemoinformatics, the solution to this problem can be seen in supporting the experimental methods with theoretical, i.e., in silico, methods which are helpful in estimating ADME/T parameters [4,5,6,7].

In silico models allow a molecule to be quickly and economically pre-tested before it is probed on animal models. An example of a computer program for estimating the parameters in question is the ADMETlab platform developed by Dong, Wang, Yao et al. [8]. Therefore, it is now aiming to reduce the time and cost of this process using both QSPR (Quantitative Structure Properties Relationships) and QSAR (Quantitative Structure Activity Relationships) studies. These methods are useful tools in the search for bioactive compounds or new drug formulations and allow the time and the costs of their development to be cut.

Recently, a large number of QSPR/QSAR models have been developed, and different programs (available on-line) based on them allow for highly accurate predictions of the ADME/T properties of bioactive compounds. The use of in silico experiments at the initial stages of drug development makes it possible to select the drug candidates with unfavorable properties even before the start of clinical tests [9,10,11,12,13].

QSAR analysis provides quantitative information relating properties of the molecule to its biological activity. QSPR models are mathematical models relating chemical structure to various physicochemical and physical properties. Many research studies have been conducted in this area in recent years [12,13,14,15,16,17,18,19,20,21,22,23,24,25,26]. Numerous published QSAR and QSPR models include lipophilicity parameters obtained by thin-layer chromatography and high-performance liquid chromatography in a reversed phase system, i.e., RP-TLC or RP-HPLC as R_MW_ and logk_w_ values, respectively or using computational approaches in the form of logP [27,28,29,30,31,32,33]. The chromatographic analysis is rapid and repeatable and until today has been suitable for the study of lipophilicity of different classes of bioorganic compounds [27,28,29,30,31,32,33]. Lipophilicity is a physicochemical parameter that has to be widely used in developing new drugs. Due to the observable importance of lipophilicity parameters and its key role in understanding the pharmacokinetic properties of drug candidates (i.e., significant influence on ADME/T properties), a big need for elaboration accurate and precise experimental as well as in silico models for the prediction of lipophilicity descriptors has arisen.

A relatively new tool in design of the drugs, i.e., in predicting their physical and physicochemical parameters including lipophilicity, is the use of different molecular descriptors such as topological indices [33,34,35,36]. To the most popular indices belong Randić indices (^0^χ, ^1^χ, ^0^χ^ν^, ^1^χ^ν^), the Balaban index (I_B_), Gutman indices (M, M^ν^) and the Wiener index (W) calculated on the basis of chemical structure of a proper bioactive molecule or predicted by means of computer programs such as Dragon as presented in previous papers [34,35,36,37,38,39,40,41].

Topological indices have been successfully correlated with various physical and physicochemical properties, such as boiling point, density, surface tension, critical pressure, critical volume and heat capacity as well evaporation heat of different compounds [42,43]. Moreover, the selected topological indices have been utilized as an important tool in modeling the lipophilicity and pharmacological activity of newer compounds as potential antiviral and anticancer drug candidates and others [44,45,46,47,48,49,50,51]. Topological indices have been correlated well with theoretical and chromatographic parameters of lipophilicity, other biologically active compounds, such as steroids, fatty acids and nicotinic acid derivatives [44,45,46,47,48,49,50,51,52]. In our recent works, we demonstrated the usefulness of selected topological indices such as Randić (^0^χ, ^0^χ^ν^, ^1^χ^ν^) and Gutman (M, M^ν^) indices in predicting logP value of two pharmaceutically important antidiabetic agents i.e., metformin and phenformin [50]. A new method for calculating logP value of naproxen based on topological indices was also proposed by Pyka and coworkers in 2013 [44].

Therefore, as a continuation of our previous paper on the comparative study of selected anti-androgenic and blood uric acid-lowering compounds [52], the aim of the current study was to calculate the following topological indices based on adjacency and distance matrix, respectively, such as Gutman (M, M^ν^), Randić (^0^χ, ^1^χ, ^0^χ^ν^, ^1^χ^ν^), Wiener (W), Rouvray–Crafford (R) and Pyka (A, ^0^B, ^1^B) for selected anti-androgenic agents: abiraterone bicalutamide, flutamide, nilutamide, leflunomide, teriflunomide, ailanthone and some hypouricemic compounds, such as allopurinol, oxypurinol and febuxostat. To the authors’ knowledge, there are no data in the literature regarding these indices for all the compounds studied. Next, the utility of newly calculated topological indices for predicting the ADME/T important physicochemical properties, such as lipophilicity descriptors (R_MW_ and logP) as well as boiling point, index of refraction, molar refractivity, polar surface area, polarizability, surface tension and molar volume of investigated compounds, was estimated. The presented correlations in the form of linear equations can be a fast and effective method for assessing the lipophilicity of tested compounds as an important component in the processes of design of their new analogues or new pharmaceutical formulations.

## 2. Results and Discussion

The topological indices calculated for the studied anti-androgens and blood uric-acid lowering compounds according to Equations (83)–(95) described in earlier papers [38,39,40,41] are listed in Table 1. This table shows the values of the following topological indices: Wiener (W), Rouvray–Crafford (R), Pyka (A, ^0^B, ^1^B), Randić (^0^χ, ^1^χ, ^0^χ^ν^, ^1^χ^ν^) and Gutman (M, M^ν^).

Analysis of the data collected in Table 1 shows the differences in the values of topological indices of the studied compounds depending on the formula used to calculate them and the structure of each of them based on which calculations for these descriptors have been carried out. Generally, the smallest values in the range of 0.2531–0.7468 and 2.3403–2.7868 show the two indices proposed by Pyka, i.e., ^1^B and ^0^B, respectively. However, the highest values of all the calculated indices are shown for the Rouvray–Crafford index (R). In this case, the R values ranging from 152.786 (for allopurinol) to 3495.225 (for bicalutamide) are observed.

**Figure 1:**
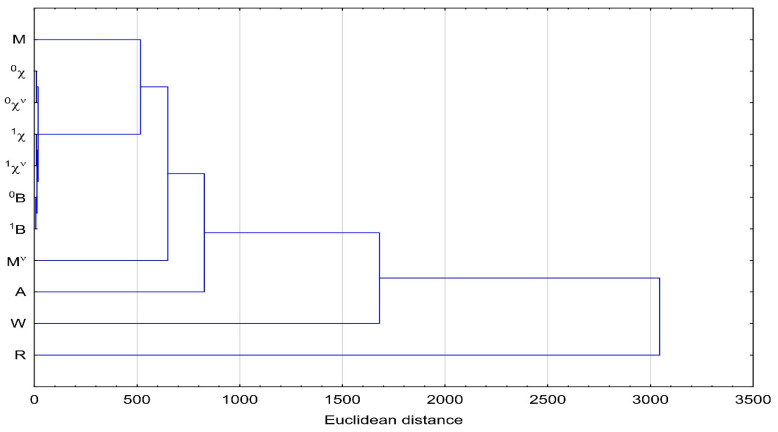
Cluster analysis of all calculated topological indices.

The similarity analysis (CA) carried out in the next stage enabled a more accurate comparison of the values of the 11 topological indices obtained in the work. Figure 1, i.e., dendrogram of similarity of all calculated topological indices, confirms that all Randić indices (^0^χ, ^1^χ, ^0^χ^ν^, ^1^χ^ν^) and Pyka indices (^0^B and ^1^B) based on adjacency and distance matrix, respectively, show the biggest similarity (the smallest Euclidean distance). The other indices do not form visible clusters. The Rouvray index (R) based on the distance matrix can be seen as the most distant from the others.

**Figure 2:**
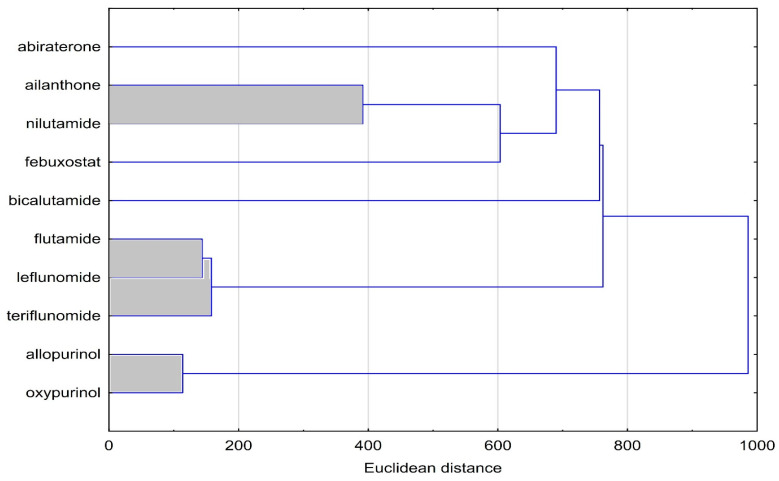
Cluster analysis of tested compounds based on calculated topological index values.

Next, Figure 2 shows the results for the cluster analysis (CA) of tested compounds based on their topological index values.

Cluster analysis presented in Figure 2 allowed the similarity between the studied bioactive substances belonging to two different pharmacological groups, namely, anti-androgenic and hypouricemic compounds, to be found. A dendrogram of similarity analysis presented in Figure 2 shows that there are three visible clusters in the figure. The first includes ailanthone and nilutamide, the second flutamide, leflunomide and teriflunomide, and the third allopurinol and oxypurinol. The similarity of the compounds that are part of the clusters results, of course, from the fact that the topological indices were calculated based on the structure of the compounds. Hence, the compounds in clusters have a similar structure. This is most evident in the pair of compounds: allopurinol and oxypurinol, and in the case of three compounds in the second cluster (flutamide, leflunomide and teriflunomide). It can be concluded that topological indices can be helpful for grouping selected compounds with anti-androgenic and hypouricemic activity. However, the results of this analysis are influenced not only by the biological activity of clustered compounds but also their chemical structure.

**Figure 3:**
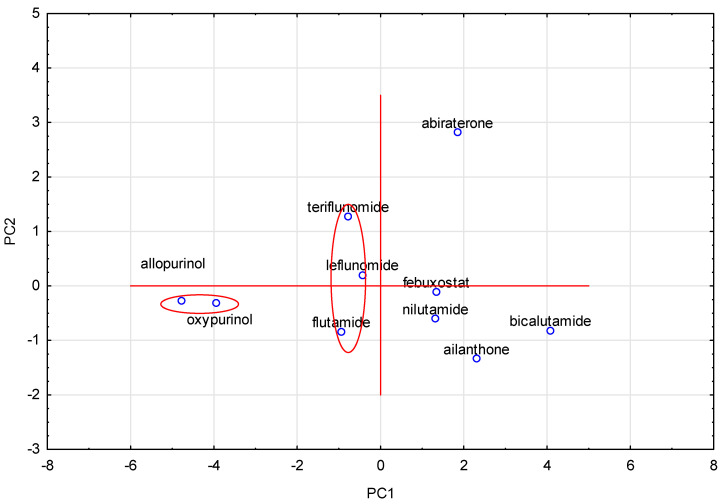
PCA analysis of studied compounds based on calculated topological index values.

Next, on the basis of the obtained results, an analysis of principal components (PCA) was also performed. All data have been reduced using the Kaiser criterion to only two principal components, for which the eigenvalues have a value greater than 1. The projection plot for these components is shown in Figure 3.

Analysis of Figure 3 confirms the results of previously performed analysis of similarities (CA), which is indicated by a marked pair of compounds, namely allopurinol and oxypurinol, and the second marked group, i.e., teriflunomide, leflunomide and flutamide with a similar chemical structure.

The aim of further steps of this work was to estimate the usefulness of newly calculated topological indices for both groups of studied compounds with anti-androgenic and hypouricemic activity, respectively, in the prediction of selected physicochemical properties of these bioactive compounds including their lipophilicity parameters, as in QSPR study. Therefore, the previously obtained parameters of lipophilicity for these compounds, by means of TLC method under various chromatographic conditions, expressed as R_MW_ values as well as the theoretical values of logP predicted with the use of different algorithms, such as AlogPs, AClogP, AlogP, MlogP, XlogP2, XlogP3, logP_KOWWIN_ and ACD/logP as was published in our earlier paper [52], were compared with these topological indices of the studied compounds. The results of the constructed correlation matrix between the topological indices of the tested compounds and theoretical or chromatographic parameters of lipophilicity in the form of logP and R_MW_ values allowed for the selection of the best linear relationships between a proper topological index of these compounds and the parameters of lipophilicity. The best linear correlations with *p* < 0.05 are presented in Table 2.

Analysis of the data presented in Table 2 shows a strong correlation between the theoretical partition coefficients expressed as AlogPs, AClogP, XlogP2 and ACD/logP (as a measure of the lipophilicity of the studied hypouricemic compounds having blood uric acid-lowering properties) and almost that of the newly calculated topological indices in this work, i.e., with Wiener, Gutman, Pyka, Rouvray–Crafford and Randic’ indices. For all obtained equations, i.e., Equations (1)–(17), the high correlation coefficient (r) is observed. The number of linear correlations between the newly calculated descriptors, i.e., topological indices and theoretical values of logP for the second group of the studied compounds, namely anti-androgens, is smaller (Equations (18)–(23)) compared to the first group. The values of the correlation coefficients are lower and negative. As it can be observed, of all the topological indices based on both the adjacency matrix and distance matrix, good correlations between the selected partition coefficients such as AlogPs, AClogP, mlogP, XlogP3, its average value (logP_avg_) and with logP_KOWWIN_, shows that only the Randić index expresses as ^1^χ (Table 2). This fact confirms the utility of index ^1^χ for predicting the theoretical value of logP of the tested compounds according to Equations (18)–(23).

Taking into account our previous study [52] on the determination of the theoretical as well as experimental, i.e., chromatographic parameters for both groups of the studied compounds as a continuation of this work, we estimated the linear relationships among the newly calculated topological indices and chromatographic parameters of lipophilicity, i.e., R_MW_ values obtained in earlier studies by means of chromatographic plates precoated with silica gel RP18F_254_, RP18WF_254_ and RP2F_254_ and three binary mobile phases consisting of ethanol–water (EtOH/H_2_O), acetonitrile–water (ACN/H_2_O) and propan-2-ol–water (P-2-ol/H_2_O) [52]. The obtained linear equations are shown in Table 3 as Equations (24)–(37). It can be observed that the best correlations for blood uric acid-lowering compounds were achieved between the chromatographic parameters of lipophilicity determined using RP18F_254_ or RP18WF_254_ plates and a proper mobile phase such as EtOH/H_2_O or ACN/H_2_O with almost all of the calculated topological indices, while there was no satisfactory correlation for R_MW_ obtained by means of RP2F_254_ plates. The best linear correlation for this group of studied compounds, i.e., with r = 0.9999, occurred in the case of Equation (30): RP18 (ACN/H_2_O) = −2.358 + 0.01248 × M^ν^. Thus, index M^ν^ can be successfully applied for the prediction of chromatographic parameters of lipophilicity (R_MW_) of these compounds obtained on RP18F_254_ plates using acetonitrile–water as the mobile phase. Other linear equations presented for this group in Table 3 also show high correlations (r > 0.99) between R_MW_ values determined using RP18F_254_ or RP18WF_254_ plates and ethanol–water or acetonitrile–water as the mobile phases and a proper topological index of all calculations in this work except Randić indices ^0^χ and ^1^χ. Therefore, the two descriptors are not suitable for assessing the lipophilicity of these compounds.

The last two equations listed in Table 3 as Equations (36) and (37) indicate linear relationships between the chromatographic parameter of lipophilicity of the second group of studied compounds, i.e., with anti-androgenic activity and topological indices of Pyka ^0^B and ^1^B. These correlations are smaller (r = −0.7581 and r = −0.8043) compared to the previous group, but can be useful to predict the R_MW_ values of the anti-androgens developed using RP18F_254_ and RP18WF_254_ plates and propan-2-ol–water Equations (36) and (37).

In further study, we tried to estimate the relationships among the newly calculated topological indices of both blood uric acid-lowering compounds and anti-androgens and other physicochemical properties of them, such as boiling point (B_P_), index of refraction (I_R_), molar refractivity (M_R_), polar surface area (P_SA_), polarizability (P), surface tension (S_T_) and molar volume (M_V_). These properties were also presented in our previous paper [52].

Table 4 shows the equations and parameters of linear correlations between the topological indices of the tested blood uric acid-lowering compounds and their physicochemical properties.

As can be seen the equations presented in Table 4, i.e., Equations (38)–(59) show strong linear relationships with high correlation coefficient in the range of 0.9978 ≤ r ≤ 1.0000 between the following physicochemical properties of studied hypouricemic compounds such as molar refractivity (M_R_), polarizability (P) and molar volume (M_V_), and all the calculated topological indices except for index Pyka ^0^B and ^1^B. The largest number of linear correlations created indices M, W, R, A, ^0^χ^ν^ and ^1^χ^ν^. Therefore, they can be successfully applied to predict the three properties of the tested substances with uric acid-lowering activity such as allopurinol, oxypurinol and febuxostat. Both Randić indices ^0^χ and ^1^χ correlate good only with molar refractivity (M_R_) and polarizability (P).

Next, Table 5 shows all linear relationships between the selected physicochemical properties and newly calculated topological indices determined for the second analyzed group, i.e., anti-androgens (abiraterone, bicalutamide, flutamide, nilutamide, leflunomide, teriflunomide, ailanthone).

The analysis of the results presented in Table 5 indicates the greatest diversification for the obtained linear correlations of the selected physicochemical properties of the tested anti-androgens and their topological indices. The number of obtained linear correlations in Equations (60)–(82) is similar to those in the previously described group. Although, these correlation equations contain more physicochemical parameters compared to the earlier group, such as boiling point (B_P_), index of refraction (I_R_), molar refractivity (M_R_), polar surface area (P_SA_), polarizability (P), surface tension (S_T_) and molar volume (M_V_). These parameters correlate well with the appropriate topological index of all those obtained in this work except for the Pyka indices A, ^0^B and ^1^B. The correlation coefficients range from r = 0.7560 (Equation (60)) to r = 0.9940 (Equation (68)). It can therefore be concluded that the newly calculated topological indices show a good predictive power for the selected physicochemical properties of the tested anti-androgens. Comparison of the obtained results to our previous studies [42,51] concerning the application of the selected topological indices for the evaluation of some physicochemical properties including the chromatographic parameter of lipophilicity (R_MW_) and theoretical partition coefficient (logP values) of some bile acids, namely, cholic, glycocholic, glycodeoxycholic, chenodeoxycholic, deoxycholic, lithocholic and glycolithocholic, shows the greatest variety of linear equations created between the topological indices and both of the lipophilicity descriptors in the current work. It was stated that the best predictive power in the estimation of the logP values for the investigated hypouricemic drugs was achieved for linear equations containing the following topological indices: M, M^ν^, ^0^χ, ^1^χ, ^0^χ^ν^, ^1^χ^ν^, W, R, A, ^0^B and ^1^B. While the successful evaluation of lipophilicity parameters from early studied bile acids enabled only the Gutman index (M^ν^) and Pyka index (C) [42,51]. The utility of the described topological indices ^0^χ^ν^, ^0^χ, ^1^χ^ν^, M and M^ν^ in the development of new calculation procedures for the logP prediction as a measure of the lipophilicity of metformin and phenformin has also been confirmed in our relatively newer work published in 2021 [50].

## 3. Materials and Methods

### 3.1. Calculation of Topological Indices

One of the most important computational techniques is topological indices derived from graph theory. Topological indices are otherwise sets of numerical descriptors that describe the molecule under investigation. The structure under study can be represented as a graph with atoms as vertices and covalent bonds as edges. The arrangement of individual atoms in a molecule is strictly determined by its topology and also its geometry, and the components in question correlate with the pharmacokinetic properties of the molecules. Topological indices can take different forms and are calculated according to the equations of their authors. To calculate the topological index, it is necessary to represent the molecule in the form of a graph. Hydrogen atoms are ignored, and the others form vertices. Each bond is an edge of the graph [38,39,40,41].

For the anti-androgens and blood uric acid-lowering compounds studied, topological indices based on the distance matrix and the adjacency matrix were calculated. These include the Wiener (W), Rouvray–Crafford (R), Pyka (A, ^0^B, ^1^B), Randić (^0^χ, ^1^χ, ^0^χ^ν^, ^1^χ^ν^) and Gutman (M, M^ν^) indices [38,39,40,41].

The indices W, R, A, ^0^B and ^1^B were calculated from the distance matrix. The elements of this matrix were calculated using the method presented by Barysz et al. [39]. The diagonal elements of the matrix can be represented by the formula:

(83) $$d_{ii}=1-6/Z_{i}$$

where Z_i_ is the atomic number of atom i.

On the other hand, those elements that do not lie on the diagonal (called “the off-diagonal”) are described by the formula:

(84) $$d_{ij}=\sum_{r}k_{k}$$

where the summation is carried out on the vertices r. The parameter k_r_ can be described by the formula:

(85) $$k_{r}=\frac{1}{b_{r}}\times \frac{36}{Z_{i}Z_{j}}$$

In the formula discussed above, br takes the value of 1 for a single bond, 2 for a double bond or 3 for a triple bond. Z_i_ and Z_j_, on the other hand, are the atomic numbers of atoms labeled as i and j, forming bond r. The values of k_r_ for typical bonds occurring most often are shown in Table 6:

The Rouvray–Crafford index (R) was calculated by summing up all elements of the distance matrix. The equation below corresponds to this as

(86) $$R=\sum_{i}d_{ii}+\sum_{ij}d_{ij}$$

Wiener and Pyka indices (W, A, ^0^B, ^1^B) were calculated according to their authors’ formulas listed below:

(87) $$W=\sum_{i}d_{ii}+\sum_{i<j}d_{ij}$$

(88) $$A=\sqrt{\sum_{l}^{n_{m}}{{\left(S_{i}\right)}_{l}}^{2}}$$

(89) $${}^{1}B=\sum_{l}^{n_{m}}{{\left(S_{i}S_{j}\right)}_{l}}^{-1/2}$$

(90) $${}^{0}B=\sum_{l}^{n_{m}}{{\left(S_{i}\right)}_{l}}^{-1/2}$$

In the above equations, l is the number identifying the subgraphs, S_i_ and S_j_ are the sums of the distances of vertex i and vertex j from all other vertices of the graph. i and j are the neighboring vertices in the subgraph. Index ^1^B also takes into account the double bond between oxygen and carbon in the carbonyl group.

Gutman index M and Randić indices ^0^χ and ^1^χ were calculated using the formulas below:

(91) $$M=\sum_{i=l}^{N}{\left(\delta_{i}\right)}^{2}$$

(92) $${}^{m}\chi =\sum_{j=l}^{n_{m}}\prod_{i=l}^{m+1}{{{(\delta }_{i})}_{j}}^{-1/2}$$

In these formulas, δ represents the degrees of vertices (δ_i_ is the i-th degree of vertices in a graph and means the number of neighboring vertices or the number of edges falling on a vertex), m takes the values 0 or 1, n_m_ represents the number of connections.

Gutman index M^ν^ and Randić indices ^0^χ^ν^ and ^1^χ^ν^ were calculated using the delta valence δ^ν^ according to the following equations:

(93) $$M^{\nu }=\sum_{i=l}^{N}{\left(\delta_{i}^{\nu }\right)}^{2}$$

(94) $${}^{m}\chi^{\nu }=\sum_{j=l}^{n_{m}}\prod_{i=l}^{m+l}{{\left(\delta_{i}^{\nu }\right)}_{j}}^{-1/2}$$

In these formulas, δ^ν^ is related to the number of valence electrons of the atom that forms the vertex of the graph. The valence delta can be represented by the equation:

(95) $$\delta^{\nu }=Z^{\nu }-h$$

where Z^ν^ is the number of valence electrons of the atomic vertex, while h is the number of hydrogen atoms associated with the vertex. The symbol ν indicates that the degree of the vertex δ was calculated in the base of Z^ν^.

### 3.2. Chromatographic Parameters of Lipophilicity (R_MW_)

The chromatographic parameters of lipophilicity (R_MW_ values) of anti-androgen compounds, such as abiraterone, bicalutamide, flutamide, nilutamide, teriflunomide, leflunomide and ailanthone and blood urea-lowering agents, i.e., allopurinol, oxypurinol and febuxostat, were published in a previous paper [52]. The methodology of Soczewiński-Wachtmeister was used to determine the chromatographic parameters of lipophilicity for the studied compounds [53]. Chromatographic analysis was performed on different adsorbents, i.e., aluminum plates precoated with silica gel RP18F_254_ and glass plates coated with silica gel RP18WF_254_ and silica gel RP2F_254_ developed with three binary mixtures consisting of ethanol, acetonitrile, propan-2-ol and water [52].

### 3.3. Calculated Parameters

Besides the chromatographic parameters of lipophilicity (R_MW_), the lipophilicity descriptors of the studied compounds were also obtained theoretically as logP (the logarithm of partition coefficient). As described in detail in our previous work [52], different calculators available online at Virtual Computational Chemistry Laboratory (http://www.vcclab.org./, accessed on 20 October 2022) and at ChemSpider (http://www.chemspider.com, accessed on 20 October 2022) have been used including fragmental methods (ACD/logP, AlogP), atom-based methods (AClogP, xlogP2, xlogP3) and atom/fragment contributions (logP_KOWWIN,_) as well as property dependent methods (AlogPs, MlogP). While the n-octanol partition coefficient, i.e., experimental values of logP (given as logP_exp_) for the selected studied compounds allopurinol, bicalutamide, flutamide, nilutamide and leflunomide were obtained from DrugBank (https://www.drugbank.com/, accessed on 20 October 2022) [52]. Other physicochemical parameters of the tested compounds, such as boiling point, density, index of refraction, molar refractivity, polarizability, polar surface area, surface tension and molar volume were from EPIWEB 4.1 program (Estimation Programs Interface Suite TM Version 4.1). All data were accessed in October 2022 [52].

### 3.4. Cluster Analysis (CA)

Cluster analysis (CA), also known as similarity analysis, is one of the most widely used statistical tool which allows for the dataset to be divided into groups (clusters) [54,55]. The goal of this process is to find similar groups of objects and to identify relationships between them next. In this work, cluster analysis of newly calculated topological indices of the studied compounds, namely, Gutman index (M, M^ν^), Randić indices (^0^χ, ^1^χ, ^0^χ^ν^, ^1^χ^ν^), Wiener index (W), Rouvray–Crafford index (R) and Pyka indices (A, ^0^B, ^1^B) as well as all the examined compounds was performed using the Statistica 13.3 program [54]. The calculations were based on Euclidean distances and single connection distances as measures of similarity between the data being compared (topological indices or studied compounds, respectively), as shown on the corresponding dendrograms. The applied method was satisfactorily used in this work to compare the tested compounds belonging to two different pharmacological groups, namely anti-androgens and hypouricemic agents.

### 3.5. Principal Component Analysis (PCA)

Principal component analysis (PCA for short) is the oldest and most powerful technique to reduce the complexity of data. Its aim is to reduce a larger set of variables into a smaller set of variables called principal components that still contains most of the information in the large set and effectively describes the variability of the system [54,55]. The PCA method is easy to compute and useful for the visualization of data in different scientific disciplines including medicine, biology and pharmacy [54,55]. The analysis of principal components of the studied compounds based on the calculated topological index values was conducted using Statistica 13.3 software. Prior to this analysis, data standardization was carried out. With this standardization, it will be possible to change the data values so they can be analyzed when they have different dimensions. The number of eigenvalues was determined based on the Kaiser’s rule and scree plot.

### 3.6. Linear Regression

Linear regression is one of the basic statistical methods in which to model a relationships between the set of variables: dependent variables (y) and one or more independent variables (x). The result is a linear regression equation that can be used to make predictions about various data such as unknown physicochemical properties of bioactive compounds including lipophilicity descriptors, which is key in the ADMET process. In order to create a simple linear model between the newly calculated topological indices based on the adjacency matrix and distance matrix, such as M, M^ν^, ^0^χ, ^1^χ, ^0^χ^ν^, ^1^χ^ν^, W, R, A, ^0^B and ^1^B and different physicochemical properties of the studied compounds including chromatographic and theoretical parameters of lipophilicity (R_MW_) and logP values (successfully obtained during our previous study by TLC method) on RP2F_254_, RP18F_254_ and RP18WF_254_ plates by mixtures: ethanol–water, acetonitrile, propan-2-ol as mobile phases and with the use of different algorithms, such as AlogPs, AClogP, AlogP, MlogP, XlogP2, XlogP3, logP_KOWWIN_ and ACD/logP, the program of Statistica 13.3 was applied [54]. The form of the obtained models was y = ax + b, where y represents a proper topological index, a represents the coefficient, b represents the intercept, x shows the lipophilicity descriptor (R_MW_/logP) or other physicochemical properties of the studied compounds, such as boiling point (B_P_), index of refraction (I_R_), molar refractivity (M_R_), polar surface area (P_SA_), polarizability (P), surface tension (S_T_) and molar volume (M_V_). These properties were also presented in our previous paper [52]. The best linear models are performed in Table 2, Table 3, Table 4 and Table 5.

## 4. Conclusions

The provided investigations concerning the calculation of different topological indices based on the adjacency matrix and distance matrix, namely Gutman (M, M^ν^), Randić (^0^χ, ^1^χ, ^0^χ^ν^, ^1^χ^ν^), Wiener (W), Rouvray–Crafford (R) and Pyka (A, ^0^B, ^1^B), for selected anti-androgenic drugs (abiraterone, bicalutamide, flutamide, nilutamide, leflunomide, teriflunomide, ailanthone) and hypouricemic compounds (allopurinol, oxypurinol, febuxostat) indicate that the newly calculated descriptors allow for simple linear regression equations to be obtained which enable a reliable value of lipophilicity parameters for the studied compounds to be obtained as an important component of the ADME/T profile and other properties such as density, boiling point, index of refraction, molar refractivity molar volume, polarizability, polar surface area and surface tension. The correlation coefficients of the created linear equations show strong relationships between certain topological indices and both the earlier determined (during previous studies) chromatographic parameters of lipophilicity (R_MW_) and theoretical logP as measures of lipophilicity for the examined compounds. However, the number of linear models obtained for predicting lipophilic parameters is greater for hypouricemic drugs compared to the second studied group, namely anti-androgens. The proposed linear models based on topological indices are fast, easy to use and low cost compared to experimental ADME/T studies. In the future, it is planned to use the topological indices presented in this paper to develop new correlations, whose equations will allow for the calculation of other important ADME/T properties of the investigated anti-androgens and hypouricemic agents such as human intestinal absorption, carcinogenicity or acute oral toxicity.

## Figures and Tables

**Table 1:** Topological indices of studied compounds based on the adjacency and distance matrix.

Compound	Topological Indices Based on Adjacency Matrix						Topological Indices Based on Distance Matrix				
	M	M^ν^	^0^χ	^1^χ	^0^χ^ν^	^1^χ^ν^	W	R	A	^0^B	^1^B
	Blood Uric Acid-Lowering Compounds										
Allopurinol	96	184	5.9309	3.6481	4.9574	2.7451	76.804	152.786	48.6479	2.5718	0.7468
Oxypurinol	102	218	6.6903	4.0423	5.3410	2.9328	101.248	201.424	61.1606	2.5845	0.6837
Febuxostat	190	342	14.499	8.3593	14.6247	6.7450	810.546	1619.431	955.8462	2.6186	0.3456
	Anti-Androgens										
Abiraterone	210	246	16.8011	1.5871	16.1181	9.4695	1447.587	2893.781	577.6900	2.4969	0.2531
Bicalutamide	287	573	19.0629	10.1215	14.7881	7.8966	1749.234	3495.225	661.7294	2.6786	0.2656
Flutamide	135	399	13.9309	7.5186	10.1154	2.0128	526.913	1051.791	246.1452	2.5929	0.3781
Nilutamide	160	462	15.1280	7.8722	11.3934	5.9729	1067.496	2132.563	786.2401	2.6346	0.3388
Leflunomide	166	402	12.5823	7.0160	9.7846	4.3595	588.241	1174.697	274.7566	2.4525	0.3471
Teriflunomide	170	298	12.8012	6.6709	9.7462	5.0397	640.855	1278.586	297.3176	2.3403	0.3003
Ailanthone	204	404	17.2760	10.9384	14.4220	9.0418	1152.250	2302.750	456.6286	2.7868	0.3619

**Table 2:** Linear correlation equations between topological indices of tested compounds and their partition coefficients.

Partition Coefficient (logP)	Topological Index	Linear Equation	Correlation Coefficient (r)	No. Eq.
Blood Uric Acid-Lowering Compounds				
AlogPs	W	AlogPs = −1.060 + 0.00599 × W	0.9970	(1)
AlogPs	R	AlogPs = −1.057 + 0.00300 × R	0.9970	(2)
AlogPs	A	AlogPs = −0.7926 + 0.00480 × A	0.9982	(3)
AClogP	W	AClogP = −0.8596 + 0.00610 × W	0.9971	(4)
AClogP	R	AClogP = −0.8570 + 0.00305 × R	0.9971	(5)
AClogP	A	AClogP = −0.5875 + 0.00489 × A	0.9983	(6)
XlogP2	M^ν^	XlogP2 = −5.144 + 0.02257 × M^ν^	0.9984	(7)
XlogP2	^0^χ	XlogP2 = −3.122 + 0.39545 × ^0^χ	0.9976	(8)
XlogP2	^1^χ	XlogP2 = −3.384 + 0.71734 × ^1^χ	0.9973	(9)
XlogP2	^1^B	XlogP2 = 5.6126 − 8.714 × ^1^B	−1.0000	(10)
ACD/logP	M	ACD/logP = −112.3 + 1.1217 × M	0.9984	(11)
ACD/logP	^1^χ	ACD/logP = −87.95 + 22.558 × ^1^χ	0.9972	(12)
ACD/logP	^0^χ^ν^	ACD/logP = −56.95 + 10.794 × ^0^χ^ν^	0.9994	(13)
ACD/logP	^1^χ^ν^	ACD/logP = −75.65 + 26.173 × ^1^χ^ν^	0.9992	(14)
ACD/logP	W	ACD/logP = −14.00 + 0.14181 × W	0.9996	(15)
ACD/logP	R	ACD/logP = −13.94 + 0.07094 × R	0.9996	(16)
ACD/logP	A	ACD/logP = −7.640 + 0.11365 × A	0.9999	(17)
Anti-Androgens				
AlogPs	^1^χ	AlogPs = 5.6093 − 0.4320 × ^1^χ	−0.8667	(18)
AClogP	^1^χ	AClogP = 5.0751 − 0.3574 × ^1^χ	−0.8910	(19)
mlogP	^1^χ	mlogP = 4.6684 − 0.2898 × ^1^χ	−0.7764	(20)
XlogP3	^1^χ	XlogP3 = 5.9902 − 0.4834 × ^1^χ	−0.8154	(21)
logP_avg_	^1^χ	logP_avg_ = 5.1783 − 0.3768 × ^1^χ	−0.8425	(22)
logP_KOWWIN_	^1^χ	logP_KOWWIN_ = 6.9299 − 0.5693 × ^1^χ	−0.9099	(23)

**Table 3:** Linear correlation equations between topological indices of the tested compounds and their R_MW_ values obtained with the use of different stationary phases (RP18F_254_ and RP18W F_254_ plates) developed by mixtures: EtOH/H_2_O, ACN/H_2_O and P-2-ol/H_2_O (ethanol–water, acetonitrile–water, propan-2-ol–water) as mobile phases.

Chromatographic Parameter of Lipophilicity (R_MW_)	Topological Index	Linear Equation	Correlation Coefficient (r)	No. Eq.
Blood Uric Acid-Lowering Compounds				
RP18 (EtOH/H_2_O)	M	RP18 (EtOH/H_2_O) = −2.520 + 0.02750 × M	0.9976	(24)
RP18 (EtOH/H_2_O)	^0^χ^ν^	RP18 (EtOH/H_2_O) = −1.163 + 0.26467 × ^0^χ^ν^	0.9989	(25)
RP18 (EtOH/H_2_O)	^1^χ^ν^	RP18 (EtOH/H_2_O) = −1.621 + 0.64167 × ^1^χ^ν^	0.9985	(26)
RP18 (EtOH/H_2_O)	W	RP18 (EtOH/H_2_O) = −0.1099 + 0.00348 × W	0.9991	(27)
RP18 (EtOH/H_2_O)	R	RP18 (EtOH/H_2_O) = −0.1085 + 0.00174 × R	0.9991	(28)
RP18 (EtOH/H_2_O)	A	RP18 (EtOH/H_2_O) = 0.04581 + 0.00279 × A	0.9997	(29)
RP18 (ACN/H_2_O)	M^ν^	RP18 (ACN/H_2_O) = −2.358 + 0.01248 × M^ν^	0.9999	(30)
RP18 (ACN/H_2_O)	^0^B	RP18 (ACN/H_2_O) = −110.3 + 42.834 × ^0^B	0.9989	(31)
RP18 (ACN/H_2_O)	^1^B	RP18 (ACN/H_2_O) = 3.5769 − 4.798 × ^1^B	−0.9974	(32)
RP18W (ACN/H_2_O)	W	RP18W (ACN/H_2_O) = −0.4364 + 0.00245 × W	0.9973	(33)
RP18W (ACN/H_2_O)	R	RP18W (ACN/H_2_O) = −0.4354 + 0.00122 × R	0.9973	(34)
RP18W (ACN/H_2_O)	A	RP18W (ACN/H_2_O) = −0.3272 + 0.00196 × A	0.9984	(35)
Anti-Androgens				
RP18 (P-2-ol/H_2_O)	^1^B	RP18 (P-2-ol/H_2_O) = 4.5928 − 7.182 × ^1^B	−0.7581	(36)
RP18W (P-2-ol/H_2_O)	^0^B	RP18W (P-2-ol/H_2_O) = 9.8338 − 3.232 × ^0^B	−0.8043	(37)

**Table 4:** Equations and parameters of linear correlations between topological indices of the tested blood uric acid-lowering compounds and their physicochemical properties, molar refractivity—M_R_; polarizability [cm^3^]—P; molar volume [cm^3^]—M_V_.

Physicochemical Parameter	Topological Index	Linear Equation	Correlation Coefficient (r)	No. Eq.
M_R_	M	M_R_ = −15.89 + 0.52076 × M	0.9997	(38)
M_R_	^0^χ	M_R_ = −0.7278 + 5.7737 × ^0^χ	0.9990	(39)
M_R_	^1^χ	M_R_ = −4.596 + 10.479 × ^1^χ	0.9992	(40)
M_R_	^0^χ^ν^	M_R_ = 9.8652 + 5.0076 × ^0^χ^ν^	1.0000	(41)
M_R_	^1^χ^ν^	M_R_ = 1.1774 + 12.144 × ^1^χ^ν^	1.0000	(42)
M_R_	W	M_R_ = 29.792 + 0.06577 × W	1.0000	(43)
M_R_	R	M_R_ = 29.820 + 0.03290 × R	1.0000	(44)
M_R_	A	M_R_ = 32.753 + 0.05268 × A	0.9997	(45)
P	M	P = −6.211 + 0.20575 × M	0.9997	(46)
P	^0^χ	P = −0.2209 + 2.2811 × ^0^χ	0.9989	(47)
P	^1^χ	P = −1.749 + 4.1401 × ^1^χ	0.9991	(48)
P	^0^χ^ν^	P = 3.9624 + 1.9786 × ^0^χ^ν^	1.0000	(49)
P	^1^χ^ν^	P = 0.53001 + 4.7984 × ^1^χ^ν^	1.0000	(50)
P	W	P = 11.836 + 0.02599 × W	1.0000	(51)
P	R	P = 11.847 + 0.01300 × R	1.0000	(52)
P	A	P = 13.006 + 0.02082 × A	0.9998	(53)
M_V_	M	M_V_ = −95.86 + 1.7703 × M	0.9978	(54)
M_V_	^0^χ^ν^	M_V_ = −8.455 + 17.039 × ^0^χ^ν^	0.9990	(55)
M_V_	^1^χ^ν^	M_V_ = −37.97 + 41.311 × ^1^χ^ν^	0.9987	(56)
M_V_	W	M_V_ = 59.330 + 0.22386 × W	0.9993	(57)
M_V_	R	M_V_ = 59.426 + 0.11199 × R	0.9993	(58)
M_V_	A	M_V_ = 69.362 + 0.17943 × A	0.9998	(59)

**Table 5:** Equations and parameters of linear correlation equations obtained between topological indices of the tested anti-androgen drugs and their physicochemical properties, where boiling point [°C]—B_P_; index of refraction—I_R_; molar refractivity—M_R_; polar surface area [A°]—P_SA_; polarizability [cm^3^]—P; surface tension [dyne/cm]—S_T_; molar volume [cm^3^]—M_V_.

Physicochemical Parameter	Topological Index	Linear Equation	Correlation Coefficient	No. Eq.
B_P_	M	B_P_ = 103.44 + 1.9858 × M	0.7560	(60)
B_P_	^0^χ	B_P_ = −286.5 + 49.958 × ^0^χ	0.9324	(61)
B_P_	^0^χ^ν^	B_P_ = 8.4148 + 38.328 × ^0^χ^ν^	0.7902	(62)
B_P_	W	B_P_ = 244.79 + 0.23084 × W	0.8231	(63)
B_P_	R	B_P_ = 245.04 + 0.11542 × R	0.8230	(64)
I_R_	^0^χ^ν^	I_R_ = 1.4038 + 0.01315 × ^0^χ^ν^	0.7992	(65)
I_R_	^1^χ^ν^	I_R_ = 1.4775 + 0.01415 × ^1^χ^ν^	0.8622	(66)
M_R_	^0^χ	M_R_ = −26.99 + 6.7763 × ^0^χ	0.8675	(67)
M_R_	^0^χ^ν^	M_R_ = −9.561 + 7.0284 × ^0^χ^ν^	0.9940	(68)
M_R_	^1^χ^ν^	M_R_ = 37.344 + 6.3639 × ^1^χ^ν^	0.9024	(69)
M_R_	W	M_R_ = 40.612 + 0.03567 × W	0.8724	(70)
M_R_	R	M_R_ = 40.636 + 0.01784 × R	0.8727	(71)
P_SA_	M^ν^	P_SA_ = −9.044 + 0.22389 × M^ν^	0.7852	(72)
P_SA_	^1^χ	P_SA_ = 11.136 + 9.3195 × ^1^χ	0.9273	(73)
P	^0^χ^ν^	P = −49.99 + 7.1096 × ^0^χ^ν^	0.7836	(74)
S_T_	^0^χ	S_T_ = −2.977 + 3.3866 × ^0^χ	0.7761	(75)
S_T_	^1^χ^ν^	S_T_ = 29.281 + 3.1633 × ^1^χ^ν^	0.8029	(76)
M_V_	M	M_V_ = 99.569 + 0.71158 × M	0.7679	(77)
M_V_	^0^χ	M_V_ = −21.71 + 16.701 × ^0^χ	0.8834	(78)
M_V_	^0^χ^ν^	M_V_ = 26.659 + 16.883 × ^0^χ^ν^	0.9865	(79)
M_V_	^1^χ^ν^	M_V_ = 143.81 + 14.571 × ^1^χ^ν^	0.8537	(80)
M_V_	W	M_V_ = 142.43 + 0.09032 × W	0.9128	(81)
M_V_	R	M_V_ = 142.49 + 0.04518 × R	0.9131	(82)

**Table 6:** Values of k_r_ for typical bonds occurring most often.

Binding Type	k_r_ Value
C-C	1.000
C-O	0.750
C=O	0.375
C=C	1.500
C-Cl	0.353
C-N	0.857
C=N	0.428

## Data Availability

Not applicable.

## Sample Availability

Samples of the compounds are available from the authors.
